# Moesin as a prognostic indicator of lung adenocarcinoma improves prognosis by enhancing immune lymphocyte infiltration

**DOI:** 10.1186/s12957-021-02229-y

**Published:** 2021-04-10

**Authors:** Yan-Qi Li, Zhi Zheng, Quan-Xing Liu, Xiao Lu, Dong Zhou, Jiao Zhang, Hong Zheng, Ji-Gang Dai

**Affiliations:** Department of Thoracic Surgery, Xinqiao Hospital, Army Medical University (Third Military Medical University), Chongqing, 400037 China

**Keywords:** Lung adenocarcinoma, Moesin, Infiltration, Inflammation, Immune lymphocytes, Poor prognosis

## Abstract

**Background:**

Ezrin-radixin-moesin (ERM) have been explored in many cancer processes. Moesin, as its component, has also been found to play an important role in the prognosis of cancer patients, tumor metastasis, drug resistance, and others. Especially in regulating the immunity, but most results came from direct studies on immune cells, there is no clear conclusion on whether moesin has similar effects in tumor cells. And moesin has certain research results in many cancers in other aspects, but there are few about moesin in lung adenocarcinoma (LUAD).

**Methods:**

We detect the expression of moesin in 82 LUAD and matched normal tissue samples by immunohistochemistry. Besides, for the pathological feature, we did a detailed statistical analysis. And with the help of various databases, we have done in-depth exploration of moesin’s ability to enhance the extent of immune lymphocyte infiltration.

**Results:**

Moesin is a poor expression in lung cancer tissues than the corresponding normal samples. And this phenomenon had a strongly associated with the prognosis and TNM stage of these LUAD patients. Moesin can enhance the infiltration of multiple immune lymphocytes in lung cancer. And this may be related to the interaction between moesin and various inflammatory molecules.

**Conclusions:**

Moesin is a newly index for the prognosis of LUAD and improves the prognosis of LUAD patients by regulating a variety of inflammation-related molecules to enhance immune lymphocytes infiltration.

## Background

Lung carcinoma is still the most common malignant tumor with the highest morbidity and mortality in China, and in recent years, LUAD has gradually developed into the highest rates in lung cancer [1]. Due to the difficulty of early diagnosis with the high risk of metastasis and recurrence, the treatment of LUAD patients is still a dilemma on clinic [2]. Therefore, to find a high sensitivity and specificity index to help the diagnosis and mechanistic studies to guide the treatment of LUAD is necessary for the current clinical situation.

Moesin is a member of the highly homologous ezrin-radixin-moesin (ERM) family of proteins that possess an N-terminal four-point-one, ezrin, radixin, moesin (FERM) domain and C-terminal actin-binding domain. Research in recent years has shown that ERM plays an important role in various cancer-related processes such as driving tumor signals, maintaining cancer cell survival and cancer invasion and metastasis [3–7]. When further study the functions of its individual proteins, it is surprising that they seem to have some undiscovered effects in regulating immunity [8, 9]. Study on moesin has shown that it is closely related to the homeostasis and self-tolerance of T cell and B cell, as well as the outflow of these cells from secondary lymphoid organs [10–12]. Studies have also shown that moesin functioned in the production of regulatory T cells (Tregs) induced by TGF-β by stabilizing type II (TβRII) TGF-β receptors on the T cell membrane [13]. In addition, a recent study pointed out that the dephosphorylation and inactivation of moesin in T cells was beneficial for the combination of T cells and APCs, thereby promoting the proliferation and activation of T cells [14]. These all suggest that how moesin regulates the body’s immune capacity is a direction that worth exploring. However, although moesin has been found to make a difference in regulating immunity came from direct studies on immune cells, there is still no clear conclusion about what changes and mechanisms that moesin will cause for immune system in tumor cells.

But it should be pointed out that moesin has been found to have unique roles in other tumor-related processes except regulating immunity. It was considered an independent prognosis factor for many tumors, like oral squamous cell carcinoma and ER-positive breast cancer [15, 16]. Besides, there found that G protein-coupled receptor kinase GRK5 can phosphorylate moesin to promote prostate cancer metastasis and MiR-200 can promote cancer cell invasion through an unclear way which depended on moesin [17, 18]. Interestingly, compared with changing the migration and invasion ability of a single cancer cell, studies have pointed out that Rab11 may trigger the collective migration of tumor cells by controlling the activity of moesin, but the more specific mechanisms still need further explored [19]. Furthermore, p53-microRNA 200-moesin axis was found to involve in the drug resistance in cancer cells, which showed the potential that regulating moesin to reverse tumor resistance [20]. In summary, these research results showed that moesin is an indicator that worth exploring in tumors, and in-depth study will be also beneficial for the tumor clinical diagnosis and treatment. But unfortunately, there was no relevant research report about moesin be found in LUAD. Therefore, it has great significance to explore the relation and reason between moesin and LUAD.

## Methods

### Western blotting

Paired tumor and normal lung tissues were dissected into 1–2 mm^3^ in 1 mL mix solution that contain radioimmunoprecipitation assay (RIPA) lysis buffer (Sangon Biotech) and Halt^TM^ Protease and Phosphatase Inhibitor Cocktail (Thermo Fisher Scientifc, Waltham, MA, USA), tissue lysate was obtained by used a glass homogenizer. Then tissue lysate was centrifuged at 12,000*g* for 5 min and supernatant was collected for western blotting. The concentrations of collection protein were measured by bicinchoninic acid (BCA) protein assay kit (Beyotime Biotechnology). The protein sample was electrophoresed on 10% sodium dodecyl sulfate-polyacrylamide gels and then transferred to an Immobilon PVDF membrane. The membrane incubated with primary antibody (Rabbit anti-moesin, ab52490, abcam; Rabbit anti-β-actin, 8457S, Cell Signaling Technology) for a night at 4 °C and then slightly shake with a horseradish peroxidase-conjugated secondary antibody (goat anti-rabbit IgG-HRP, sc-2004, Santa Cruz Biotechnology). The bands were soaked with chemiluminescence detection buffer (Takara, Shiga, Japan) and its intensity was detected by the corresponding software.

### Reverse transcription and quantitative real-time PCR

Total RNA reverse transcribed by using PrimeScript™RT reagent Kit with gDNA Eraser (TAKARA, Japan). The mRNA expression quantitative analysis of moesin was used to explore the difference between the lung adenocarcinoma specimens and the corresponding para-carcinoma tissues. The PCR reaction system (25 μl) included 0.5 μl of each primer (moesin and GAPDH), 12.5 μl SYBR®Pre-mix Ex Taq TM II 2× (TAKARA, Japan), 10.5 μl ddH_2_O, and 1 μl cDNA. The process of PCR was denaturation at 95 °C for 30 s and then amplification for 40 cycles contain 15 s at 95 °C and 50 s at 60 °C, for each end of the circulation, there was a fluorescence acquisition. Using Illumina Eco software (Illumina, San Diego, USA) to detect the optical density of each PCR band. Primer sequence for moesin: FW: 5′-AAGGACCGCAGTGAGGAGGAA

C-3′, RV: 5′-CTTGGACTCATCTCTGGCATTGGC-3′. Primer sequence for GADPH: FW: 5′-GGACCTGACCTGCCGTCTAG-3′, RV: 5′-CCTGCTTCACCACCTTCTTG A-3′.

### Clinical patients

The tissue microarray contains 90 LUAD samples and 82 matched para-carcinoma tissues. These patients were diagnosed at the Xinqiao Hospital, Army Military Medical University. A detailed follow-up was done from 2009-2014 and continued to 2017. However, 9 patients were lost follow-up in the process. All the patients had not received radiotherapy or chemotherapy before surgery. No patients with a history of immune system disorders.

### Immunohistochemistry and scoring

The immunohistochemistry (IHC) assay of moesin was carried out with the Dako Envision FLEX+ system. After these tissue specimens went through section, dewaxing, and antigen retrieval, they wound incubated with the antibody (Rabbit anti-moesin, ab52490, abcam) overnight at 4 °C and then with a HRP-conjugated antibody (Dako) at 37 °C. At these slices were stained with 3-diaminobenzidine (DAB) for 2 min. The following were the scoring rules for the expression intensity: 0, almost no staining; 1, mild staining; 2, medium staining; 3, strong staining. For the positive cell ratio: 0, 0–20%; 1, 20–40%; 2, 40–60%; 3, 60–80%; 4, 80–100%. The final scores were the product of the two scores (0, 1, 2 ,3, 4, 6, 8, 9, or 12). Scores 0, 1, 2, 3, 4, and 6 are low expression, and 8, 9, and 12 are high expression. And statistical X-tile software was used to analyse the result.

### Statistical analysis

All data were analysed using the IBM SPSS Statistics 19. Student’s *t* test was performed to compare the expression of moesin between lung cancer and normal tissues. The association between moesin expression and clinicopathological characteristics were evaluated by *χ*^2^ tests. Univariate and multivariate analysis were based on Cox proportional hazard regression models. Kaplan-Meier was used to explore the association between the moesin expression and the prognosis of lung cancer patients. *P* < 0.05 was considered to indicate a statistically significant difference.

## Data mining and analysis

We downloaded the expression data of moesin from GSE32863 in NCBI GEO, TCGA, Oncomine and GEPIA database, then using R package (version 4.0.3) to observe its expression status in lung carcinoma and normal para-carcinoma tissues, all the results were tested with Student’s *t* test. For the LUAD patients, we performed a chi-test for all pathological features with the matched expression level of moesin. As for the prognosis of these patients, we conducted survival analysis using Kaplan-Meier method with moesin as the factor. Univariate and multivariate analysis were be used to detect the significance of moesin in the prognosis for LUAD patients. Data on the relationship between moesin and the degree of infiltration for various immune lymphocytes were from the TISIDB database. moesin co-expression molecules and degree scores were from coexpedia database, and the protein interaction network diagram was with the help of Cytoscape (version 3.7.1).

## Results

### Moesin is poor expression in lung carcinoma

**Fig. 1:**
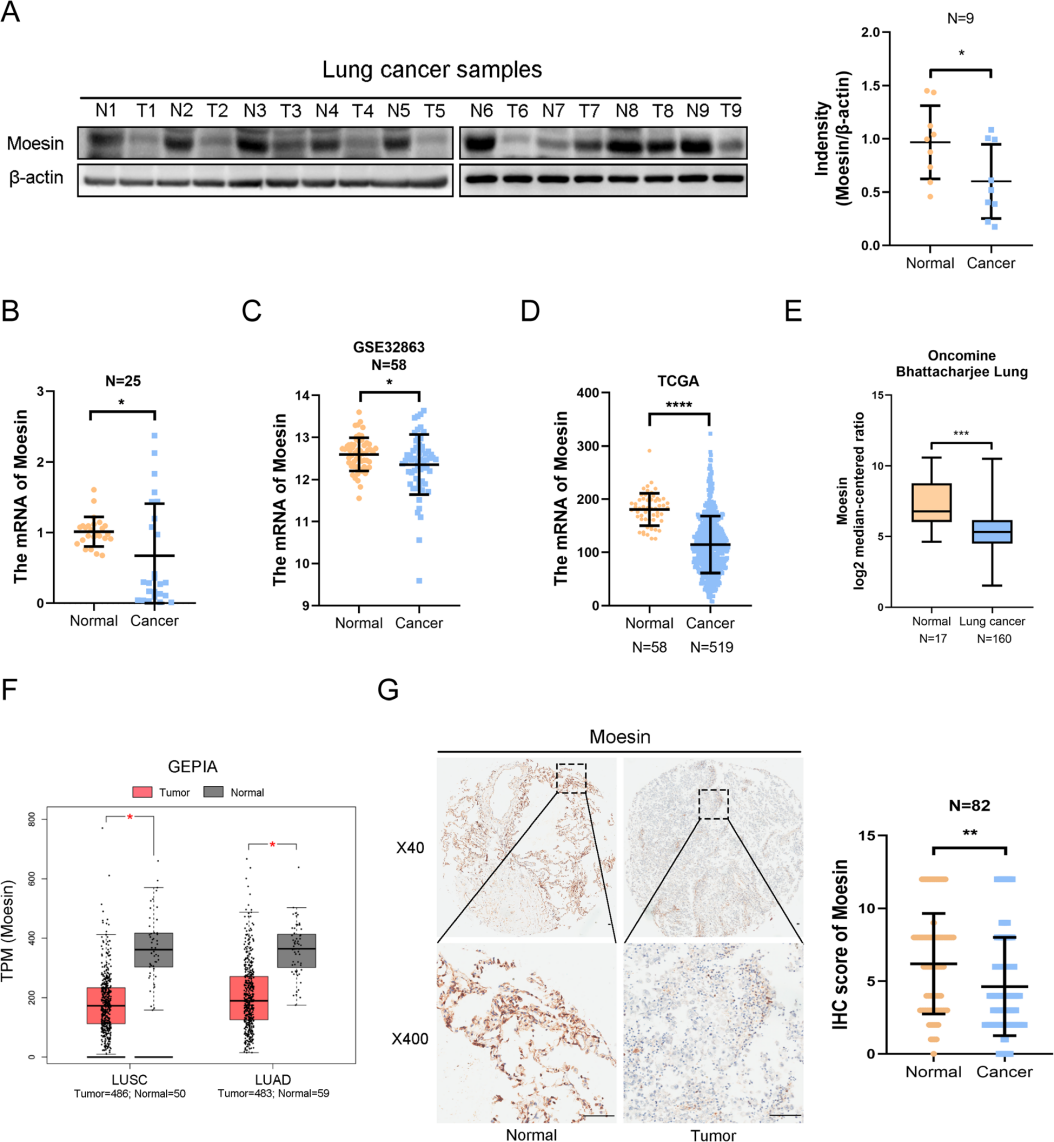
The expression status of moesin is poor in human LUAD specimens compared to the matched para-carcinoma tissues. **a**, **b** The expression level of protein and mRNA about moesin in eight LAC patients with the carcinoma and the adjacent normal tissues. **c** Comparison of the mRNA of moesin in 58 normal lung tissues and 58 lung carcinoma samples from GEO database. **d** The analysis of the mRNA of moesin in TCGA database (normal lung tissues = 58, lung carcinoma specimens = 519). **e** The analysis of the mRNA of moesin in Oncomine database. **f** The analysis of the mRNA of moesin in GEPIA database. **g** The IHC results of moesin in 82 pairs of LAC patients and results of the IHC scores about the expression level of moesin that normal tissues compared to the carcinoma samples

In order to have a profound exploration about the expression level of moesin in lung cancer, we first explore its expression status in nine lung carcinoma samples with the matched para-carcinoma tissue. Whether the western blotting including its gray scale statistical results (Fig. 1a) or qPCR (Fig. 1b) indicated that both protein and mRNA of moesin is low-expressed in lung cancer tissue. To further confirm the reliability of the results, we analyzed the expression level of moesin in GEO (Fig. 1c), TCGA (Fig. 1d), Oncomine (Fig. 1e), and GEPIA (Fig. 1f) database by data mining and the results stay the same with our previous studies. In addition, we detected the protein level of moesin in 82 pairs of lung carcinoma specimens and adjacent tissues, the results showed that moesin is mainly settled on the membrane, and obviously, the para-carcinoma has a higher level (43.9%, 36/82) than in the LUAD tissues (20%, 18/90) (Fig. 1g, Table 1). Therefore, we found that moesin has a lower expression level in lung cancer than in para-carcinoma tissues.

**Table 1:** Moesin expression in LUAD and adjacent normal mucosa

	Moesin (low)	Moesin (high)	*P* value
Carcinoma	72	18	*P* = 0.001
Adjacent normal tissue	46	36	

### Moesin can be an index related to the prognosis of LUAD patients

**Fig. 2:**
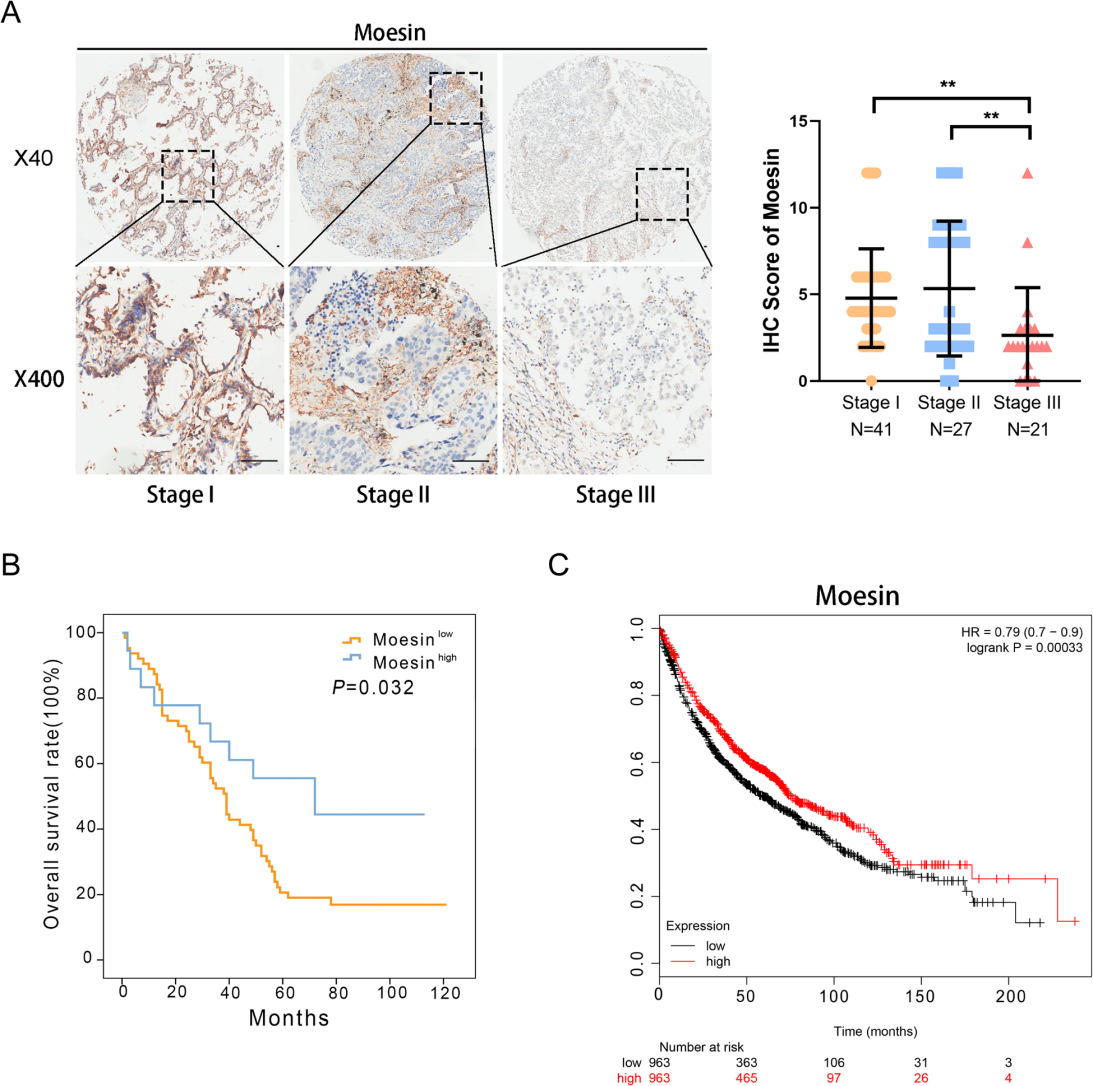
Low level of moesin is strongly related with poor prognosis. **a** IHC result of moesin in different TNM stages and statistical analysis about the relevance of moesin level with different TNM stages. **b** Kaplan-Meier analysis of these LUAD patients with the status of moesin (*P* = 0.032). **c** The result of survival analysis in Kaplan-Meier Plotter database when regarding moesin as the factor in LUAD patients

To identify whether the expression of moesin is associate with the clinical features of LUAD patients, and the clinicopathological data of these LUAD patients were shown in Table 2. Subsequently, we analyzed these data with statistical methods, and the results (Table 3) indicated that patients with high TNM stage seemed a greater probability to have a poor expression of moesin (Fig. 2a). Furthermore, in survival analysis, the LUAD patients with lower level of moesin showed a poor overall survival rate (Fig. 2b, *P* = 0.032). The data from the Kaplan-Meier Plotter database demonstrated the same phenomena (Fig. 2c, *P* < 0.001). More directly, the univariate analysis displayed the expression of moesin can be a unique indicator for the prognosis of LUAD patients (HR = 0.471; 95% CI 0.231–0.960; *P*=0.023, Table 4). All in all, these results strongly suggest that moesin can be deemed a prognostic factor for LUAD patients.

**Table 2:** The clinical features of the LUAD specimens used in this study

Feature	WHO grade		
	I (*n* = 9)	II (*n* = 66)	III (*n* = 13)
Gender			
Male	2	34	13
Female	7	32	0
Age at diagnosis			
< 60	4	29	5
≥ 60	5	37	8
*T* stage			
*T*_1–2_	7	54	10
*T*_3–4_	2	14	3
*N* stage			
*N*_*x*_	2	12	1
*N*_0_	6	25	5
*N*_1–3_	0	32	7
TNM stage			
I	5	31	5
II	5	18	4
III	0	17	4
Location			
Left	6	27	6
Right	3	38	7

**Table 3:** The relationship between moesin expression and clinicopathological features of LUAD patients

Feature	Moesin		*P* value
	High (*n* = 18)	Low (*n* = 72)	
Gender			*P* = 0.034
Male	6 (33.33%)	44 (61.11%)	
Female	12 (66.67%)	28 (38.89%)	
Age at diagnosis			*P* = 0.596
< 60	9 (50.00%)	31 (43.06%)	
≥ 60	9 (50.00%)	41 (56.94%)	
Location			*P* = 0.671
Left	7 (38.89%)	32 (44.44%)	
Right	11 (61.11%)	40 (55.56%)	
*T* stage			*P* = 0.039
*T*_1–2_	11 (61.11%)	60 (83.33%)	
*T*_3–4_	7 (38.89%)	12 (16.67%)	
*N* stage			*P* = 0.028
*N*_0_	11 (73.33%)	25 (41.67%)	
*N*_1–3_	4 (26.67%)	35 (58.33%)	
TNM stage			*P* = 0.023
I	4 (22.22%)	37 (52.11%)	
II + III	14 (77.78%)	34 (47.89%)	
Histological grade			*P* = 0.309
Well	3 (16.67%)	6 (8.57%)	
Moderate	11 (61.11%)	55 (78.57%)	
Poor	4 (22.22%)	9 (12.86%)

**Table 4:** Univariate and multivariate analysis for overall survival in LUAD

Factors	Univariate		Multivariate	
	HR (95% CI)	*P* value	HR (95% CI)	*P* value
Gender	1.288	0.326	1.009	0.977
	(0.777–2.133)		(0.558–1.824)	
Age	1.023	0.930	0.812	0.446
	(0.612–1.711)		(0.475–1.388)	
Location	1.084	0.757	1.016	0.955
	(0.650–1.809)		(0.586–1.762)	
Moesin expression	0.471	0.038	0.424	0.023
	(0.231–0.960)		(0.203–0.887)	
Grade	1.672	0.066	1.945	0.064
	(0.966–2.897)		(0.962–3.935)	
TNM stage	1.891	<0.001	1.899	<0.001
	(1.349–2.649)		(1.356–2.658)	

### Moesin can enhance the infiltration for a series of immune lymphocytes

**Fig. 3:**
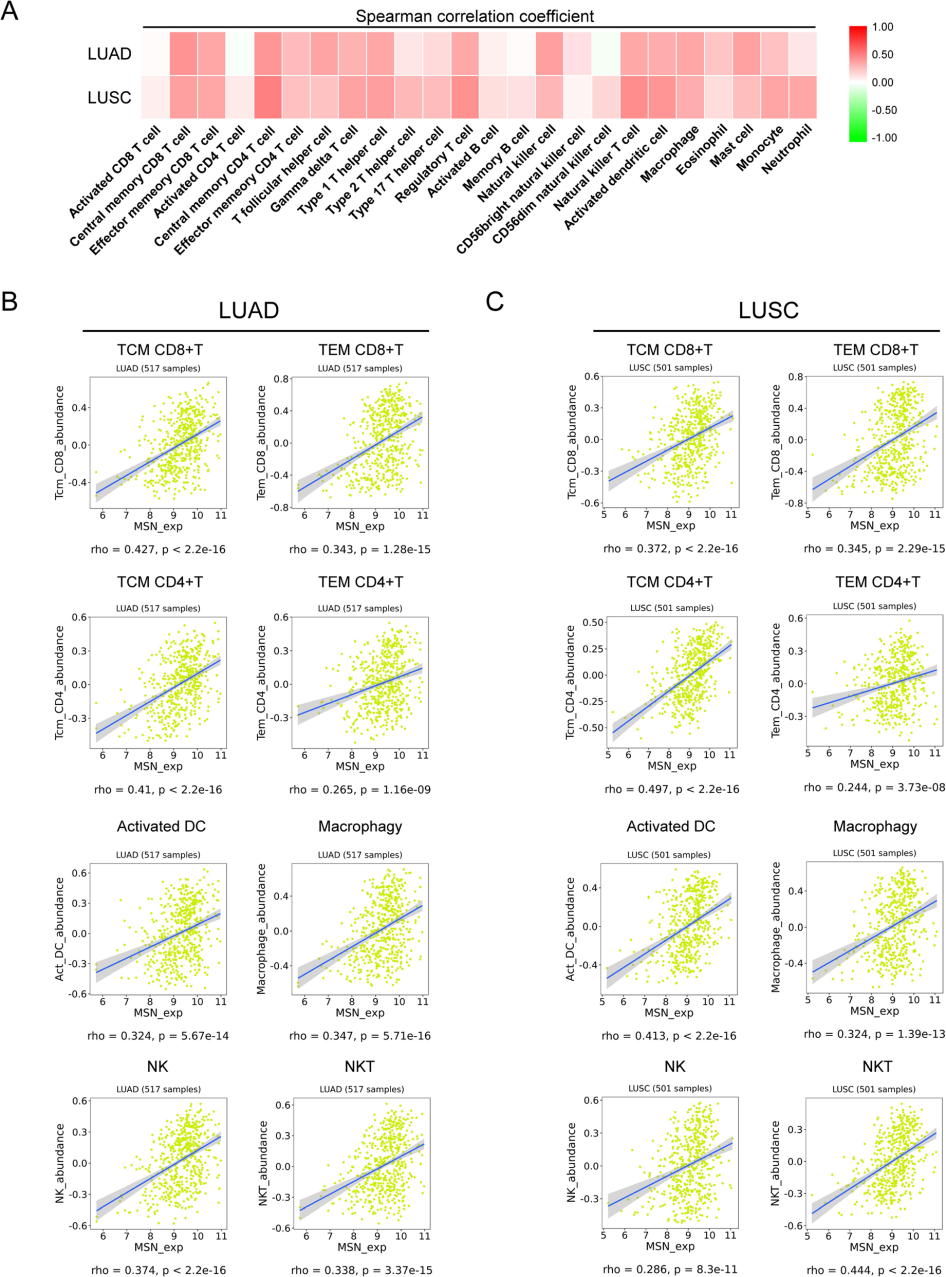
Moesin promotes the infiltration for a series of immune lymphocytes in LUAD and LUSC. **a** The heat map between moesin and the degree of infiltration for various immune lymphocytes in TISIDB database. **b** The expression of moesin and the degree of infiltration for various immune lymphocytes were significantly positively correlated in LUAD. **c** The expression of moesin and the degree of infiltration for various immune lymphocytes were significantly positively correlated in LUSC

Next, we explored the possible mechanism for high expression of moesin to improve the prognosis of lung cancer patients. As studies in recent years have shown that moesin has immune modulatory functions in immune cells and it is still unclear whether moesin has similar abilities in tumor cells, these prompted us to be interested in whether moesin expressed in lung cancer tumor cells could regulate anti-tumor immunity. To this end, we made a preliminary analysis by the TISIDB database. Surprisingly, the results showed that whether in LUAD or lung squamous cell carcinoma (LUSC), moesin promotes the infiltration for a variety of immune cells including CD4+ T cells, CD8+ T cells, and DC cells (Fig. 3a). Further analysis showed that the expression level of moesin protein was significantly related to the degree of infiltration for these immune cells (Fig. 3b, c). These results fully indicate that promoting immune lymphocyte infiltration was the mechanism of moesin.

### Moesin regulates inflammation-related molecules to promote immune lymphocyte infiltration

**Fig. 4:**
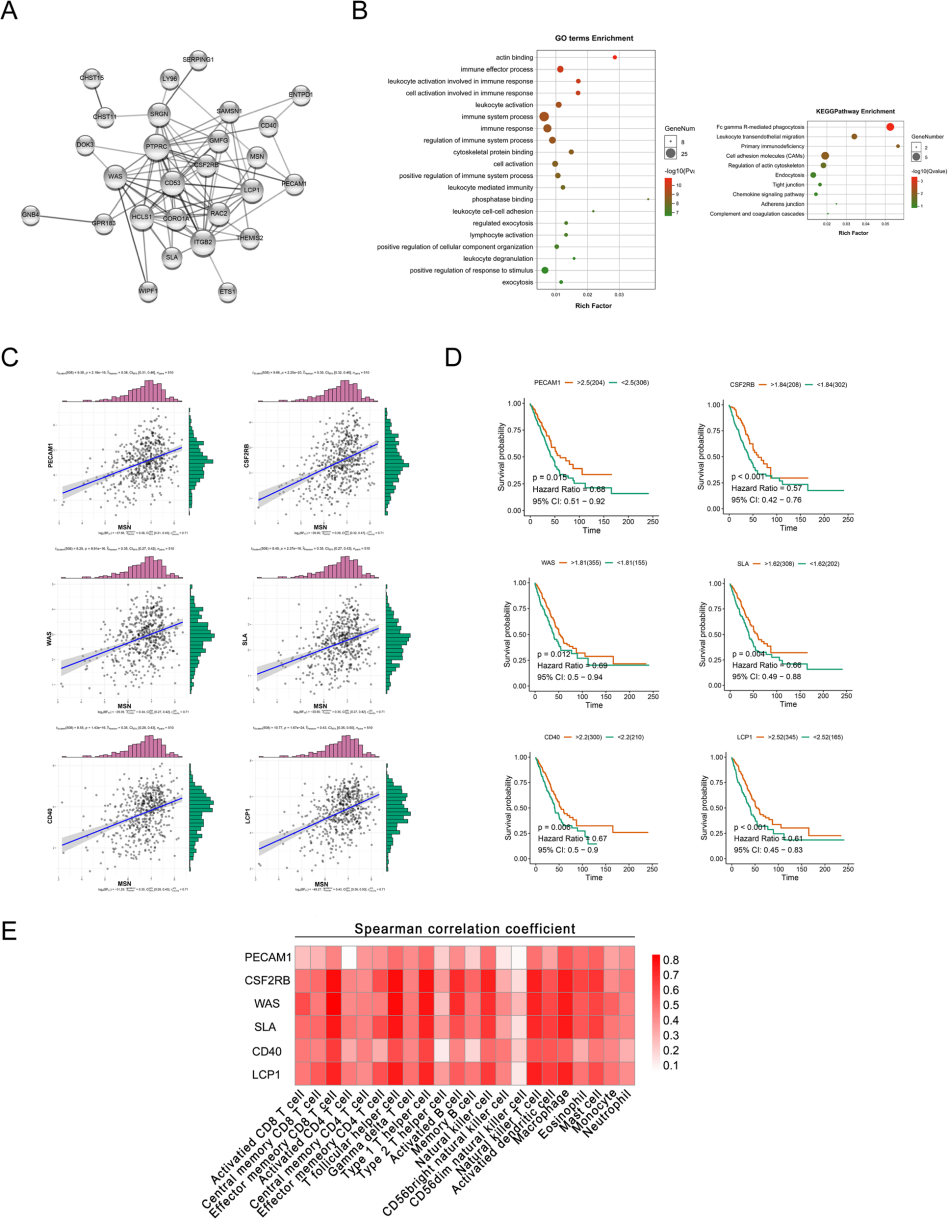
Moesin enhances immune lymphocyte infiltration by regulating a variety of inflammatory molecules. **a** Network diagram of protein interaction between moesin and inflammatory molecules with a co-expression score greater than 2. **b** GO enrichment and KEGG function enrichment results of moesin with these inflammatory molecules. **c** The expression correlation for moesin with PECAM1, CSF2RB, WAS, SLA, CD40, LCP1 in LUAD from TCGA database. **d** The relationship between the 6 genes and the prognosis of LUAD patients in TCGA. **e** Changes in the degree of immune lymphocyte infiltration caused by the 6 genes in LUAD from TISIDB database

To further explored the specific mechanism of moesin promoting immune lymphocyte infiltration, we detected the coexpression degree of moesin with a variety of inflammation-related molecules in the coexpedia database and selected molecules with a score greater than 2 for enrichment (Table 5). The results showed that moesin has an obvious interaction relationship with these inflammation-related molecules (Fig. 4a), and importantly the results of GO enrichment and KEGG enrichment indicated that they mainly play a role in immune-related processes in NSCLC cells (Fig. 4b). Further analysis for the expression data of LUAD patients in the TCGA database showed that moesin was significantly positively correlated with the expression of PECAM1, CSF2RB, WAS, SLA, CD40, and LCP1 (Fig. 4c). Meanwhile, the high expression of the 6 genes can significantly improve the prognosis of patients (Fig. 4d). And like moesin, the 6 genes can also indeed promote the infiltration of multiple immune lymphocytes (Fig. 4e). These suggest that moesin regulates the expression of a variety of inflammation-related molecules to promote immune lymphatic infiltration and thus improves the prognosis of lung cancer patients.

**Table 5:** Co-expressed genes with MSN

Gene symbol and name	LLS scores
PECAM1	4.012
RAB31	3.434
PPP1R18	2.778
ENTPD1	2.749
CSF2RB	2.704
RBMS1	2.692
ITGB2	2.661
QKI	2.658
PTPRC	2.654
CHST15	2.642
ST3GAL1	2.641
RASSF2	2.621
WIPF1	2.601
THEMIS2	2.567
CD53	2.551
RAC2	2.520
HCLS1	2.479
SRGN	2.479
CHST11	2.473
HLX	2.445
WAS	2.375
PLEKHO1	2.331
SLA	2.295
DOK3	2.281
RFTN1	2.262
GNB4	2.249
ETS1	2.227
SERPING1	2.214
GMFG	2.203
ASAP1	2.184
GPR183	2.157
SAMSN1	2.155
FAM129A	2.153
LY96	2.135
CD40	2.080
ROBO1	2.066
FAM49A	2.020
CORO1A	2.015
LCP1	2.011

## Discussion

For the low overall survival rate of LUAD in clinical because of its high recurrence ratio [21]. To find a highly specificity indicator for the poor prognosis and explore the mechanism to help the clinical treatment have important implications. Hereon, we found that moesin is obviously low-expressed in LUAD tissues than the matched para-carcinoma samples. Also, our results indicated that this phenomenon has an intense relation with the poor prognosis in clinical of LUAD. Further statistical analysis suggested that moesin can be used as a novel independent factor for the prognosis of LUAD patients.

For the specific mechanism of high expressed moesin to improve the prognosis of LUAD patients, in view of the fact that moesin has been found to regulate the body's own immunity in immune cells, we thought it may also have a similar effect in tumor cells. In fact, moesin has been found to be significantly upregulated in breast cancer, prostate cancer, pancreatic cancer, and other cancers [17, 22, 23], but there were few reports on changes the upregulation will cause in cancer. Rituximab, which binds CD20 on B cells, is widely used clinically to treat B cell malignancies and current researches showed that its anti-cancer ability mainly relies on the ADCC effect of NK cells [8, 24, 25]. A specific study pointed out that when rituximab blocks CD20, moesin will be selectively recruited to the CD20 cap, leading to polarization of cancer cells, and this polarization increased the frequency of NK cells killing target cells through ADCC by 60% compared with the non-polarization [26]. What is more, our results further showed that in lung cancer, moesin may not only promote the infiltration of NK cells. With the increasing of moesin expression, the degree of infiltration for various immune lymphocytes including central memory T (TEM), central memory T (TCM), activated DC, and NKT cells increases significantly. And it is worth noting that moesin promotes the infiltration of TCM cells, which may indicate that moesin can indeed maintain the body’s own immunity against tumor cells for a long time to improve the prognosis of cancer patients [27]. In general, these indicate that increasing the infiltration of immune lymphocytes by changing the polarity of cancer cells is only one mechanism, a more complete mechanism remains to be studied. However, there are still something interesting, a study in breast cancer indicated that the phosphorylation of moesin was necessary for programmed cell death-Ligand 1 (PD-L1) to stabilize on the cell membrane surface and silencing moesin can promote T cell activation in vitro and in vivo [28]. This suggests that in different cancers, the role of moesin may be different, research on different cancer models is conducive to the formulation of personalized clinical treatment plans.

For the fact that the activation of the body’s immune capacity is usually accompanied by an inflammatory response, we have made a preliminary exploration on whether moesin promotes the infiltration of immune lymphocytes by regulating inflammation-related signals. We initial queried the coexpression degree for moesin with a variety of inflammatory molecules in the coexpedia database and selected molecules with a coexpression score greater than 2 for follow analysis. The protein interaction network diagram showed that there was indeed a strong connection between moesin and these molecules, while GO enrichment and KEGG enrichment more directly indicated that they do have an important role in promoting the activation and migration of immune lymphocytes. This fully proved that moesin can improve the prognosis of lung cancer patients by regulating a variety of inflammatory molecules. We then further screened these inflammatory molecules, what is interesting is that we found that the 6 inflammatory molecules, PECAM1, CSF2RB, WAS, SLA, CD40, and LCP1, were not only highly positively correlated with moesin in expression, but also significantly improved the prognosis for LUAD patients in clinic. And the 6 molecules can also enhance the infiltration for a variety of immune lymphocytes like moesin, but there is no report on how moesin with the 6 molecules enhance the degree of immune lymphocyte infiltration in cancer. Specifically, the current researches showed that PECAM1 is a cell adhesion molecule required for leukocyte migration across the endothelium, and CSF2RB is a high-affinity receptor for granulocyte macrophage colony-stimulating factor [29]. SLA, CD40, LCP1, WAS may play an important role in the activation, migration, and positive sorting of T cells [30, 31]. These may be a good hint for the subsequent study about the specific mechanism of moesin with these inflammatory molecules, but research still not be limited to these directions.

Besides, recent studies have shown that moesin may also play a role in tumor drug resistance. Inhibiting the expression of moesin can reduce the expression of p-gp and BCRP proteins [20, 32] and reduce the efflux function of these proteins [33]. A more specific study pointed out that the reduction of moesin expression is mainly to reduce the expression of p-gp in the membrane, indicating that moesin may be a molecule required for p-gp to be anchored in the membrane, but the specific mechanism still needs to be further explored [34]. With clinical application value, moesin silencing restores the sensitivity to Doxorubicin of breast cancer cells [20]. Another study also pointed out that reducing the expression of moesin can increase the accumulation of methotrexate in cancer cells [35]. Interestingly, our study showed that high expression of moesin is beneficial for lung cancer patients. However, for drug resistance, current research showed that inhibiting moesin expression is related to reversing drug resistance. The connection and mechanism behind this are worthy of our further exploration in the future.

## Conclusions

In conclusion, we found that in LUAD, the expression of moesin in cancer tissues was significantly lower than that in normal tissues, and this phenomenon was strongly correlated with the poor prognosis of patients. At the same time, our analysis showed that moesin might be a molecular marker to predict the prognosis of LUAD to guide clinical diagnosis and treatment. Further studies have shown that moesin may enhance the infiltration of immune lymphocytes in cancer by regulating a variety of inflammatory molecules, thereby improving the prognosis of LUAD patients. Our data provide a new insight into the treatment and development mechanism of human LUAD.

## Data Availability

The data sets during and/or analyzed during the current study are available from the corresponding author on reasonable request

## Abbreviations

ERM: Ezrin-radixin-moesin
LUAD: Lung adenocarcinoma
FERM: Four-point-one, ezrin, radixin, moesin
Tregs: Regulatory T cells
TβRII: Type II TGF-β receptors
LUSC: Lung squamous cell carcinoma
TEM: Central memory T
TCM: Central memory T
